# A Mafia mouse model for inherited retinal degeneration

**Published:** 2013-09

Goldmann-Favre syndrome is an inherited eye disorder in which vision is weakened due to progressive retinal degeneration. Studies in both human patients and retinal degeneration mouse models have suggested that microglial cells play a role in pathogenesis; however, the underlying mechanism is unknown. In this study, Stephen Tsang and colleagues describe a novel mouse model in which the expression of circulating bone-marrow-derived microglial cells can be temporally and specifically controlled using the Mafia transgene. They report that ablation of the microglial cell population accelerates retinal degeneration, concomitant with an increase in the expression of certain inflammatory cytokines. These findings implicate inflammation as a key contributory factor in Goldmann-Favre syndrome and suggest potential targets for the development of therapies relevant to this and related neurodegenerative disorders. **Page 1113**

